# The Rapid Test Based on *Leishmania infantum* Chimeric rK28 Protein Improves the Diagnosis of Canine Visceral Leishmaniasis by Reducing the Detection of False-Positive Dogs

**DOI:** 10.1371/journal.pntd.0004333

**Published:** 2016-01-05

**Authors:** Deborah Bittencourt Mothé Fraga, Luciano Vasconcellos Pacheco, Lairton Souza Borja, Pétala Gardênia da Silva Estrela Tuy, Leila Andrade Bastos, Manuela da Silva Solcà, Leila Denise Alves Ferreira Amorim, Patrícia Sampaio Tavares Veras

**Affiliations:** 1 Laboratório de Patologia e Biointervenção, Centro de Pesquisas Gonçalo Moniz, FIOCRUZ, Salvador, Bahia, Brazil; 2 Departamento de Medicina Veterinária Preventiva e Produção Animal, Escola de Medicina Veterinária e Zootecnia, Universidade Federal da Bahia, Salvador, Bahia, Brazil; 3 Instituto de Ciência e Tecnologia de Doenças Tropicais, INCT-DT, Bahia, Brazil; 4 Instituto de Matemática, Departamento de Estatística, Universidade Federal da Bahia, Bahia, Brazil; Barcelona Institute for Global Health, SPAIN

## Abstract

Visceral Leishmaniasis (VL) has spread to many urban centers worldwide. Dogs are considered the main reservoir of VL, because canine cases often precede the occurrence of human cases. Detection and euthanasia of serologically positive dogs is one of the primary VL control measures utilized in some countries, including Brazil. Using accurate diagnostic tests can minimize one undesirable consequence of this measure, culling false-positive dogs, and reduce the maintenance of false-negative dogs in endemic areas. In December 2011, the Brazilian Ministry of Health replaced the ELISA (EIE CVL) screening method and Indirect Immunofluorescence Test (IFI CVL) confirmatory method with a new protocol using the rapid DPP CVL screening test and EIE CVL confirmatory test. A study of diagnostic accuracy of these two protocols was done by comparing their performance using serum samples collected from a random sample of 780 dogs in an endemic area of VL. All samples were evaluated by culture and real time PCR; 766 out of the 780 dogs were tested using the previous protocol (IFI CVL + EIE CVL) and all 780 were tested using the current protocol (DPP CVL + EIE CVL). Performances of both diagnostic protocols were evaluated using a latent class variable as the gold standard. The current protocol had a higher specificity (0.98 vs. 0.95) and PPV (0.83 vs. 0.70) than the previous protocol, although sensitivity of these two protocols was similar (0.73). When tested using sera from asymptomatic animals, the current protocol had a much higher PPV (0.63 vs. 0.40) than the previous protocol (although the sensitivity of either protocol was the same, 0.71). Considering a range of theoretical CVL prevalences, the projected PPVs were higher for the current protocol than for the previous protocol for each theoretical prevalence value. The findings presented herein show that the current protocol performed better than previous protocol primarily by reducing false-positive results.

## Author Summary

Visceral Leishmaniasis (VL) is a major public health problem. Its control is based on detection and culling of positive dogs, treatment of human cases and vector control. Canine cases often precede the occurrence of human cases; hence, disease control in dogs is important. Use of accurate diagnostic tests is required to avoid culling false-positive dogs and to minimize the number of false-negative dogs that are maintained in endemic areas. In December 2011, the Brazilian Ministry of Health changed the diagnostic protocol for canine VL (CVL). In the present study, the accuracy of this current protocol was compared to the previous one using serum samples of 780 dogs from an endemic area of VL. The findings revealed that the current protocol performed better than the previous protocol primarily by reducing false-positive results. Considering different theoretical prevalence values, the current protocol misdiagnosed fewer dogs than the previous one.

## Introduction

Visceral leishmaniasis (VL) is a major public health problem worldwide. This disease in Brazil and Europe is caused by the protozoan parasite *Leishmania infantum*, which is transmitted to humans by the bite of sandflies from the genus *Lutzomyia* [1]. Dogs are considered the main reservoir of urban VL since: i) these animals harbor high parasitism in skin that offers a high capacity of parasite transmission to sandflies, ii) humans and dogs coexist in close proximity and iii) canine cases generally precede the occurrence of VL in humans [1–4].

The identification and euthanasia of serologically positive dogs is one of the primary VL control strategies recommended by the governments of some countries, such as Brazil. The use of accurate diagnostic tests for canine VL (CVL) can reduce failures on VL control program by minimizing maintenance of false-negative animals and culling of false-positive dogs that impact on euthanasia controversial measure, subsequently, decreasing dog owners’ compliance and society disagreement. More accurate tests could also reduce the number of false-negative dogs that are maintained in endemic areas [4].

CVL is typically diagnosed by parasitological, serological and molecular tests. In December 2011, the program of the Brazilian Ministry of Health for monitoring and control of leishmaniasis replaced the enzyme-linked immunosorbent assay (EIE CVL) screening method and the indirect immunofluorescence assay (IFI CVL) confirmatory test with a new serodiagnostic protocol for CVL composed of the Dual Path Platform (DPP CVL) screening test and the EIE CVL confirmatory test [5]. The evaluation of sensitivity and specificity revealed low values for previous protocol that detects infection by determining seropositivity in dogs. This low performance is probably due to undesirable preservation of blood samples normally collected onto filter papers. This simple procedure for sample collection is performed easily and facilitates sample storage and transportation. However, it often submits the biological specimens to stress conditions that might damage samples and lead to unreliable test results [2,6–8]. Additionally, the low sensitivity and specificity offered by the old protocol can be explained by further reasons: i) both screening EIE CVL and confirmatory IFI CVL tests have been performed using blood samples that were collected in endemic areas and then sent to reference laboratories, where the tests were performed, ii) EIE CVL and IFI CVL tests are time-consuming techniques, whereas IFI CVL has an additional difficulty to be standardized and interpreted depending on the ability of the observer to detect the antigen-antibody reaction by fluorescence microscope. This may lead to misinterpretation of the results and may compromise IFI CVL reproducibility in different laboratories.

DPP CVL is a rapid test based on a multi-epitope, recombinant chimeric protein (rK28) resulted from fusion of *L*. *infantum* genes: k9, single repeat units of k39 and k26 [9] that has been adopted as the screening method in a new protocol established by the Brazilian government. DPP CVL rapid test is an immunochromatographic assay that offers several advantages: i) rK28 was proven to provide very high levels of sensitivity and specificity for canine VL [9], ii) DPP CVL has a great potential for facilitating faster decision, since it is a point-of-care screening test that gives result within 15 minutes, iii) DPP CVL in association with the confirmatory test EIE CVL give results within 15 days, in comparison to previous protocol (EIE CVL + IFI CVL) that results were only liberated after a lengthy time interval that varied from one to two months. Thus, the incorporation of this rapid test into the current protocol accelerates the implementation of the control measures in endemic areas. In addition, this procedure uses only small blood samples and does not require specialized equipment and supplies [10].

The use of tests presenting low accuracy has serious epidemiological consequences: false-negative dogs are undetected thereby maintaining the parasite life cycle in endemic areas, and detection of false-positive dogs results in excessive dog culling. The lack of a reliable gold standard test for CVL hinders the assessment of diagnostic protocol performance and can result in misinterpretation of diagnostic test accuracy [11–16]. Indeed, although the common used gold-standard, culturing of *L*. *infantum*, is highly specific, its low sensitivity [17] hampers the evaluation of other diagnostic techniques.

In light of this limitation, latent class analysis (LCA) has been shown to be a valuable alternative to the classical validation approach of using parasitological methods as gold standards [18,19]. LCA is based on the theory that the observed results of different imperfect tests for the same disease are influenced by a latent common variable that cannot be directly measured, but can reflect accurately the true disease status. Previous studies employing LCA have accurately assessed serological [20–24] and molecular [12,25] diagnostic methods.

Despite the advantages of DPP CVL [10,14,26,27] for CVL diagnosis, few studies have assessed its performance [28,29]. To the best of our knowledge, the present study is an initial attempt designed to compare the accuracy of the current (DPP CVL and EIE CVL) and previous protocol (EIE CVL and IFI CVL) for CVL diagnosis employing a latent class variable as the reference standard. Serum samples were obtained during a cross-sectional study performed in an endemic area for VL in Brazil.

## Methods

### Ethics Statement

All experimental procedures involving dogs were carried out according to the Brazilian Federal Law on Animal Experimentation (Law no. 11794), the guidelines for animal research established by the Oswaldo Cruz Foundation (FIOCRUZ) and the Brazilian Ministry of Health Manual for the Surveillance and Control of VL [4]. The Institutional Review Board approved the present study for Animal Experimentation (CEUA, protocol no. 015/2009). Dog owners who agreed to participate in the study signed a Free, Prior and Informed Consent (FPIC) form.

### Study Area

A cross-sectional study was conducted in the municipality of Camaçari, located in the State of Bahia in Northeastern Brazil. Using district sketches of households throughout 36 districts in Camaçari obtained from the Zoonosis Control Center, a sample of domiciled dogs was randomly selected, during the years of 2011 and 2012. The sample size was calculated using Epi Info 3.5.1 (The Centers for Disease Control and Prevention—CDC, USA) based on estimates of the canine population (15,820 dogs) derived from an anti-rabies vaccination campaign and an expected CVL prevalence of 20% (5% margin of error, 95% confidence interval).

### Sampling

Dogs were classified as asymptomatic or symptomatic based on the presence or absence of the following clinical signs: emaciation, alopecia, anemia, conjunctivitis, dehydration, dermatitis, erosion, ulcerations, lymphadenopathy, and onychogryphosis. They were classified as asymptomatic when presented 0 until 3 signs or symptomatic when presented more than 3 signs. Blood and splenic aspirate samples were obtained for CVL diagnosis from each dog at the same time. Blood was collected by venipuncture in sterile tubes to obtain serum. All serum samples were stored at -20°C until serological testing. Splenic aspirate samples were obtained using a puncture technique previously described by Barrouin-Melo and collaborators (2006) [30], and modified by Solcà and collaborators (2014) for ultrasound-guided collection. All 780 splenic samples were evaluated by culture and real time PCR; 766 out of the 780 serum samples were tested using the previous protocol (IFI CVL + EIE CVL) and all 780 were tested using the current protocol (DPP CVL + EIE CVL) (S1 Fig).

### Parasitological Testing

Splenic aspirate samples were cultivated in Novy-Mac Neal-Nicolle (NNN) medium supplemented with 20% FBS (Fetal Bovine Serum, Gibco BRL, New York, USA) and 100 μg/mL of gentamicin. The cultures were maintained at 24°C for four weeks and examined weekly for the presence of parasites [31].

### Serological Tests

All serological diagnostic test kits for CVL (DPP CVL, EIE CVL and IFI CVL Bio-Manguinhos) were used in accordance with manufacturer’s recommendations.

### DNA Extraction

DNA was extracted from splenic aspirate samples using DNeasy Blood & Tissue kit from Qiagen (Hilden, Germany), in accordance with manufacturer’s recommendations. DNA concentrations were determined using a digital spectrophotometer (Nanodrop—ND-1000 Thermo Scientific, Wilmington, USA), then aliquoted at a concentration of 30 ng/μL and stored at -20°C until real time PCR amplification.

### Real Time PCR

DNA extracted from splenic aspirate samples was amplified using real time PCR technique, in accordance with the protocol established by Francino and collaborators (2006) [32] and modified by Solcà and collaborators (2014). Control samples were added in all of the real time PCR experiments. As positive controls were used splenic aspirate samples from two dogs that had previously been identified in an endemic area as positive for *Leishmania* infection and as negative controls were employed splenic aspirates of two healthy dogs from the municipality of Pelotas, Rio Grande do Sul, Brazil, an area non-endemic for CVL.

### Statistical Analysis

All test readers executing and reading the index tests had prior training and great experience in CVL diagnosis. All diagnostic testing was carried out under blinded conditions, which means that test readers interpreted the results obtained from each diagnostic technique for a given sample without knowledge of the other tests’ results. The interpretation of the results using the previous and current diagnostic protocols classified dogs as positive when both tests (screening and confirmatory) presented positive results. Epi Info 3.5.1 (The Centers for Disease Control and Prevention—CDC, Atlanta, USA) and STATA 12.0 (StataCorp LP, Texas, USA) software programs were used to analyze results.

LCA was performed to define a latent class variable to evaluate the accuracy of the diagnostic tests and employed as previously described in Solcà and collaborators (2014). Latent variable modeling used the results of the following diagnostic techniques as indictor variables: serological (EIE CVL, DPP CVL and IFI CVL Bio-Manguinhos), parasitological (culture of splenic samples), and molecular (real time PCR of splenic aspirate) tests. We chose a two-class latent class model based on goodness of fit criteria, such as the Akaike information criterion (AIC) and Bayes information criterion (BIC). We also used the Lo-Mendel-Rubin test and the entropy for model evaluation [33]. MPlus version 5 software was used to implement LCA [34].

The performance of the diagnostic tests and protocols was estimated using the latent class variable as the reference standard. Diagnostic performance was calculated in 2 x 2 contingency tables of positive and negative test results, using the command diagt in Stata. We determined specificity, positive predictive values (PPV), negative predictive values (NPV) and diagnostic accuracy with 95% exact binomial confidence intervals (CI). Diagnostic accuracy was calculated as the number of true positive + number of true negative/total number of tested serum samples. Differences among diagnostic protocols regarding their performance (sensitivity and specificity) were assessed using McNemar chi-square test (*p*-value < 0.05), for all dogs and for two categories of disease status based on symptomatology. The number of animals considered as false negative and false positive was also calculated for each of the diagnostic techniques evaluated, considering as true positive those dogs that were positive according to the latent class variable.

## Results

**Fig 1:**
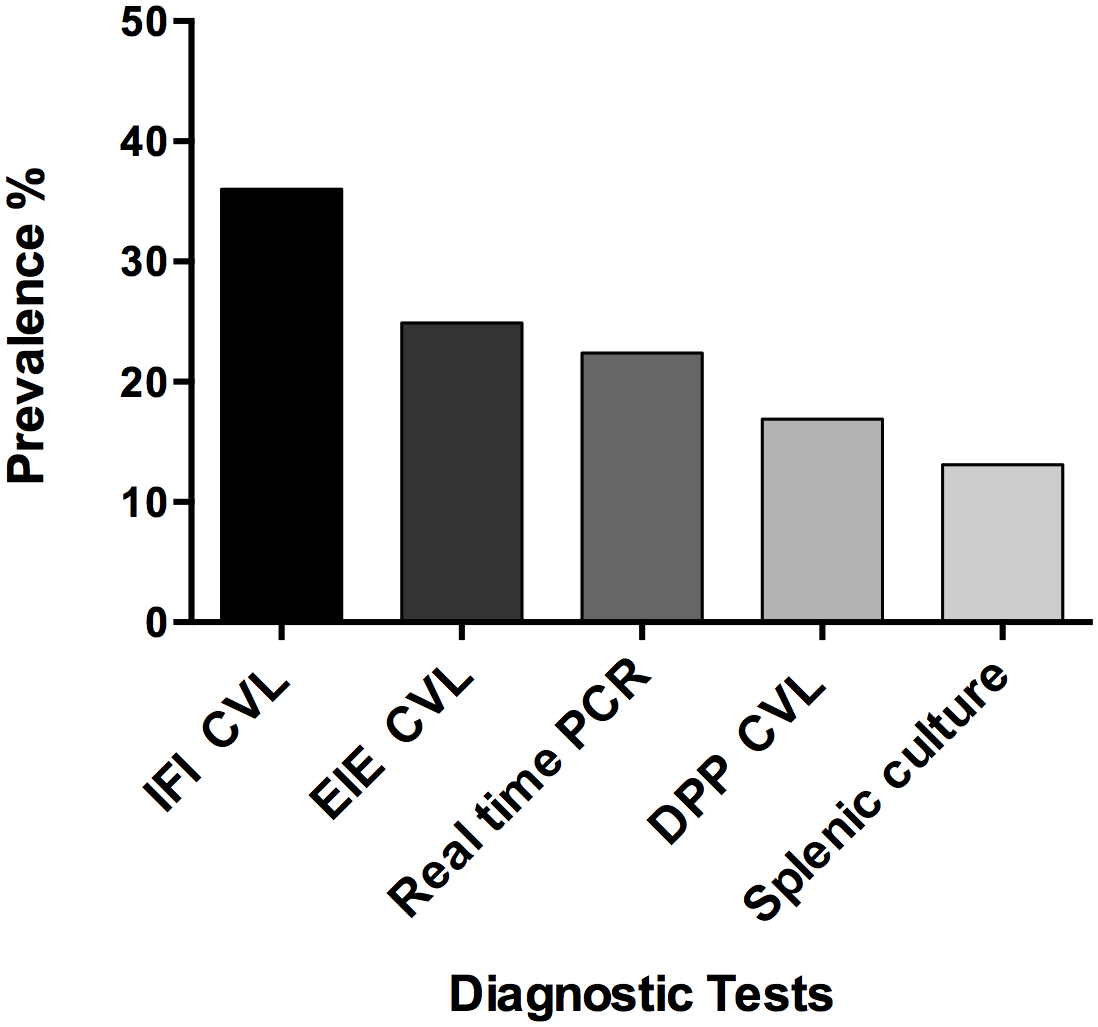
Percent of positive results of five CVL diagnostic tests performed on canine sera samples from an endemic area of Camaçari.

From April 2011 until July 2012, 780 dogs pure and mixed-breed with estimated ages from 1 to 10 years old, were enrolled in the study. According to the presence of clinical signs of CVL, 47.8% dogs were asymptomatics and 54.2% symptomatics. Five diagnostic tests were used to determine the proportion that tested positive in this random population. The IFI CVL yielded the highest percentage of positivity (36%), whereas the splenic aspirate culture yielded the lowest percentage of positivity (13.1%). Among the remaining tests, the EIE CVL, real time PCR and DPP CVL tests were positive in 24.9%, 22.4% and 16.9% of the dogs, respectively (Fig 1 and Table 1).

**Table 1:** Prevalence of latent classes and conditional probabilities according to the LCA model for CVL diagnoses.

		Latent classes	
Technique	Frequency Positive n = 780 (%)	Positive n = 110 (14.1%)	Negative n = 670 (85.9%)
		Conditional probabilities (%)	
**IFI CVL**	36.0	85.9	26.6
**EIE CVL**	24.9	79.2	15.8
**Real time PCR**	22.4	96.2	10.1
**DPP CVL**	16.9	84.1	5.6
**Culture**	13.1	88.1	0.0

Using LCA, 14.1% of the 780 dogs were classified as positive (Table 1). Evaluation of LCA entropy showed that a high accuracy in the classification of dogs by LCA was achieved, with value of 0.97. *A posteriori* average probabilities that dogs were properly classified in the latent classes "positive" and "negative" were, respectively, 95% and 99%. Moreover, the test of Lo-Mendel-Rubin indicated that the model with two classes produced better results than that with only one class (*p* < 0.01). These results are supported by the analysis of AIC and BIC (AIC = 3025.996, BIC = 3077.249).

The real time PCR and culture techniques yielded the highest sensitivities, 0.97 and 0.90, respectively, when the latent class variable served as the reference standard. Among the three serological tests, IFI CVL and DPP CVL had the highest sensitivity (0.86) and EIE CVL (0.79) (Table 2). Regarding specificity, culture was found to be the most specific (1.00), followed by DPP CVL (0.94), then real time PCR (0.90), EIE CVL (0.84) and IFI CVL (0.73).

**Table 2:** Performance of diagnostic tests considering the latent class variable as the gold standard.

Diagnostic tests	Sensitivity	Specificity	PPV	NPV
**DPP CVL**	0.86 (0.78–0.92)	0.94 (0.92–0.97)	0.71 (0.63–0.79)	0.98 (0.96–0.99)
**EIE CVL**	0.79 (0.70–0.86)	0.84 (0.81–0.87)	0.45 (0.38–0.52)	0.96 (0.94–0.98)
**IFI CVL**	0.86 (0.78–0.92)	0.73 (0.69–0.77)	0.37 (0.31–0.43)	0.97 (0.95–0.98)
**Real time PCR**	0.97(0.92–0.99)	0.90 (0.87–0.92)	0.61 (0.54–0.68)	1.00(0.99–1.00)
**Culture**	0.90 (0.83–0.95)	1.00 (0.99–1.00)	1.00 (0.96–1.00)	0.98 (0.97–0.99)

When the latent class variable was considered as the reference test, the PPV of culture was 1.00. Among the other four techniques, DPP CVL had the highest PPV (0.71), followed by real time PCR (0.61), EIE CVL (0.45) and IFI CVL (0.37). Likewise, among serological tests, DPP CVL (0.98), followed by IFI CVL (0.97) and EIE CVL (0.96) showed the highest NPV (Table 2).

The measures of diagnostic accuracy of the current diagnostic protocol were then compared to those of the previous protocol (Table 3). Both protocols had equally high sensitivity (>0.72; McNemar’s chi-square test, *p* = 0.051600) and NPV (0.96), whereas the new protocol consistently had a higher specificity (>0.97, *p* = 0.0078) and PPV (>0.83). The diagnostic accuracy was higher when current diagnostic protocol was compared to the previous protocol (0.94 vs. 0.92).

**Table 3:** Performance of current and previous protocols for CVL diagnosis, considering the latent class variable as the gold standard.

Diagnostic tests		Sensitivity	Specificity*	PPV	NPV	Accuracy
**Previous protocol**	**EIE CVL + IFI CVL (Asymptomatic dogs)**	0.71 (0.42–0.92)	0.96 (0.93–0.98)	0.40 (0.21–0.61)	0.99 (0.97–1.00)	0.95
	**EIE CVL + IFI CVL (Symptomatic dogs)**	0.74 (0.64–0.82)	0.94 (0.90–0.96)	0.79 (0.69–0.87)	0.92 (0.88–0.95)	0.89
	**EIE CVL + IFI CVL (Total)**	0.73 (0.64–0.81)	0.95 (0.93–0.96)	0.70 (0.61–0.78)	0.96 (0.94–0.97)	0.92
**Current protocol**	**DPP CVL + EIE CVL (Asymptomatic dogs)**	0.71 (0.42–0.92)	0.98 (0.96–0.99)	0.63 (0.35–0.85)	0.99 (0.97–1.00)	0.97
	**DPP CVL + EIE CVL (Symptomatic dogs)**	0.73 (0.63–0.82)	0.97 (0.94–0.98)	0.88 (0.78–0.94)	0.92 (0.89–0.95)	0.91
	**DPP CVL + EIE CVL (Total)**	0.73 (0.63–0.81)	0.98 (0.96–0.99)	0.83 (0.74–0.90)	0.96 (0.94–0.97)	0.94

*The specificity of previous and current protocol was statically different, based on McNemar test (p = 0.0078).

Comparing the performance of current protocol (DPP CVL + EIE CVL) to that of DPP CVL alone revealed that sensitivity showed higher value for DPP CVL (0.86) than that for the current protocol (0.73), although PPV showed a slight lower value for DPP CVL (0.71) compared to PPV for the current protocol (0.83).

When the dogs were categorized according to the presence of clinical signs of CVL (Table 3), the sensitivity of both diagnostic protocols was similar in asymptomatic and symptomatic dogs. However, in symptomatic dogs, the new protocol had higher specificity and PPV (0.97 and 0.88, respectively) than the previous protocol (0.94 and 0.79, respectively). In addition, in asymptomatic dogs, the PPV of the current protocol was significantly higher, by 22.5%, than that of the previous protocol (*p* = 0.0078). Also, difference was observed in diagnostic accuracy of protocols when they were used in symptomatic dogs (0.91 vs. 0.89) and asymptomatic dogs (0.97 vs. 0.95). To generalize the better performance of current protocol to other settings, the PPV and NPV were calculated for the current and previous protocol accordingly to different theoretical values of CVL prevalence (Table 4). For each estimated prevalence value, the current protocol was estimated to yield higher PPVs, ranging from 0.23 to 0.99, whereas the projected PPVs for the previous protocol ranged from 0.13 to 0.98. Regarding NPV, both protocols yielded similar projected values, ranging from 0.47 to 1.00.

**Table 4:** Estimates of PPV and NPV of current and previous protocols for CVL diagnosis, considering the latent class variable as the gold standard, by theoretical values of CVL prevalence.

	Previous protocol		Current protocol	
Prevalence (%)	PPV	NPV	PPV	NPV
**1**	0.13	1.00	0.23	1.00
**5**	0.43	0.99	0.62	0.99
**10**	0.61	0.97	0.77	0.97
**15**	0.71	0.95	0.84	0.95
**20**	0.78	0.93	0.88	0.94
**30**	0.86	0.89	0.93	0.89
**50**	0.93	0.78	0.97	0.78
**80**	0.98	0.47	0.99	0.47

## Discussion

The present study primarily demonstrated that the DPP CVL + EIE CVL protocol, in comparison with the EIE CVL + IFI CVL protocol, performed better for the serodiagnosis of CVL. The adoption of this new protocol offered several advantages, due to inclusion of the rapid DPP CVL screening test, which can be performed easily and quickly and does not require specialized equipment and personnel [27,28].

Several authors previously discussed that the lack of a perfect gold standard test for CVL hampers the evaluation of diagnostic tests for CVL [12,13]. Previous studies have proven that LCA is effective for evaluating diagnostic tests’ performance [12,20,21,35–38]. Herein, using a latent class variable as the reference standard, we were able to comprehensively compare two protocols for serodiagnosis of CVL using serum samples collected from 780 randomly selected dogs from an endemic area of VL. The use of LCA had an additional advantage: we were able to evaluate the performance of both real time PCR and culture. Very few studies have evaluated the performance of real time PCR, and most studies that evaluated the performance of CVL diagnostic techniques used culture as the gold standard [10,14,26,28]. Using LCA, we found that real time PCR and culture were the most sensitive techniques. Among the serological tests evaluated, DPP CVL had the best performance. Although IFI CVL had the highest sensitivity, it was the least specific, as previously described by de Santis and collaborators (2013) and Laurenti and collaborators (2014). Regarding the performance measure that has epidemiological relevance, the PPV, the DPP CVL had the highest PPV among the serological tests evaluated as previously described by da Silva and collaborators (2013).

In addition to evaluating each individual test for CVL diagnosis, we compared the performance of previous and current protocols employed in Brazil. The usefulness of protocols was evaluated by determining PPVs and NPVs of each protocol. The better individual performance of the DPP CVL was reflected in the 13% higher PPV of the current protocol for CVL detection compared to the previous protocol. Both protocols yielded a NPV of 0.96, suggesting that when these protocols have negative results it is highly probable that serum are from dogs that are actually uninfected. By contrast, the current diagnostic protocol provides a greater PPV (0.83) than that of the previous protocol (0.70), indicating that the current protocol provides a greater level of assertiveness in diagnosing positive dogs. Although the new protocol showed a higher specificity and PPV than the previous one, the sensitivity is still limited (around 0.73) in both protocols, meaning that the maintenance of false-negative dogs in endemic areas still represents a public health concern and more efforts should be done to try to find out better protocols or new antigens to reduce the maintenance of infected dogs in areas of zoonotic transmission.

Considering questions rose about the wisdom to diagnose CVL using DPP CVL + EIE CVL instead of DPP CVL alone, the comparison of performances showed that a higher sensitivity value (0.86) and lower PPV (0.71) for DPP CVL compared to DPP CVL + EIE CVL (0.73 and 0.83, respectively) that might cause detection of false positive dogs. Mostly to avoid this, a confirmatory test, EIE CVL, has been associated to DPP CVL in the current protocol.”

When current protocol is applied for diagnosing asymptomatic and symptomatic dogs, it showed similar performance for sensitivity (0.71, 0.73) and specificity (0.98, 0.97), respectively. While, the level of NPV (0.99) was greater, the level of PPV (0.63) was much lower for asymptomatic dogs in comparison to NPV (0.92) and PPV (0.88) for symptomatic animals. In accordance to this results, Otranto and collaborators (2009) showed that recently exposed or newly infected dogs might not be detected by serological tests, since these false-negative animals do not seroconvert soon after infection or they may develop a cellular type of immune response that are not detected using serological tests. In addition to this difficulty, no appropriate gold standard for *Leishmania* infection detection in asymptomatic dogs was established, highlighting the necessity for the development of new tests to improve diagnosis of asymptomatic dog.

Across a range of plausible prevalence, the theoretical expectation for PPV varied among 0.13 to 0.98 for previous protocol, and 0.23 to 0.99 for current protocol. PPV and NPV of a diagnostic test are known to be influenced by the prevalence of a given disease in a population. Thus, as disease becomes more prevalent the probability of subjects to test positive in diagnostic tests will be higher among sick individuals. In the present study, the analysis using different theoretical prevalence revealed that the current protocol has high performance irrespective of disease prevalence. In accordance, higher PPVs provided by DPP CVL + EIE CVL for diagnosing CVL have additional advantages since in endemic countries, regardless of the prevalence of CVL, the current protocol compared to previous one would better discriminate truly uninfected dogs from those that have risky to be infected.

In summary, our findings show that the current protocol for diagnosis of CVL implemented in Brazil has an excellent accuracy (0.91 for symptomatic dogs and 0.97 for asymptomatic), due to its greater specificity values and PPV. Because of the simplicity of test procedures and rapidity of results, the data presented herein strongly support the idea that the introduction of DPP CVL into the diagnostic CVL protocol contribute to improve CVL diagnosis that can have consequent effects that impact positively on disease control.

## Supporting Information

S1 Checklist Standards for the Reporting of Diagnostic Accuracy Studies (STARD) checklist for reporting of studies of diagnostic accuracies. (DOC)

S1 Fig STARD flowchart. Standards for the Reporting of Diagnostic Accuracy Studies (STARD) description of the experimental design to calculate accuracy of CVL serodiagnostic protocols.
(TIFF)
