# Supplementary figures and images for: The Rapid Test Based on Leishmania infantum Chimeric rK28 Protein Improves the Diagnosis of Canine Visceral Leishmaniasis by Reducing the Detection of False-Positive Dogs
Source: PLoS Negl Trop Dis. 2016 Jan 5;10(1):e0004333. doi: 10.1371/journal.pntd.0004333 (PMC4701173; doi:10.1371/journal.pntd.0004333)

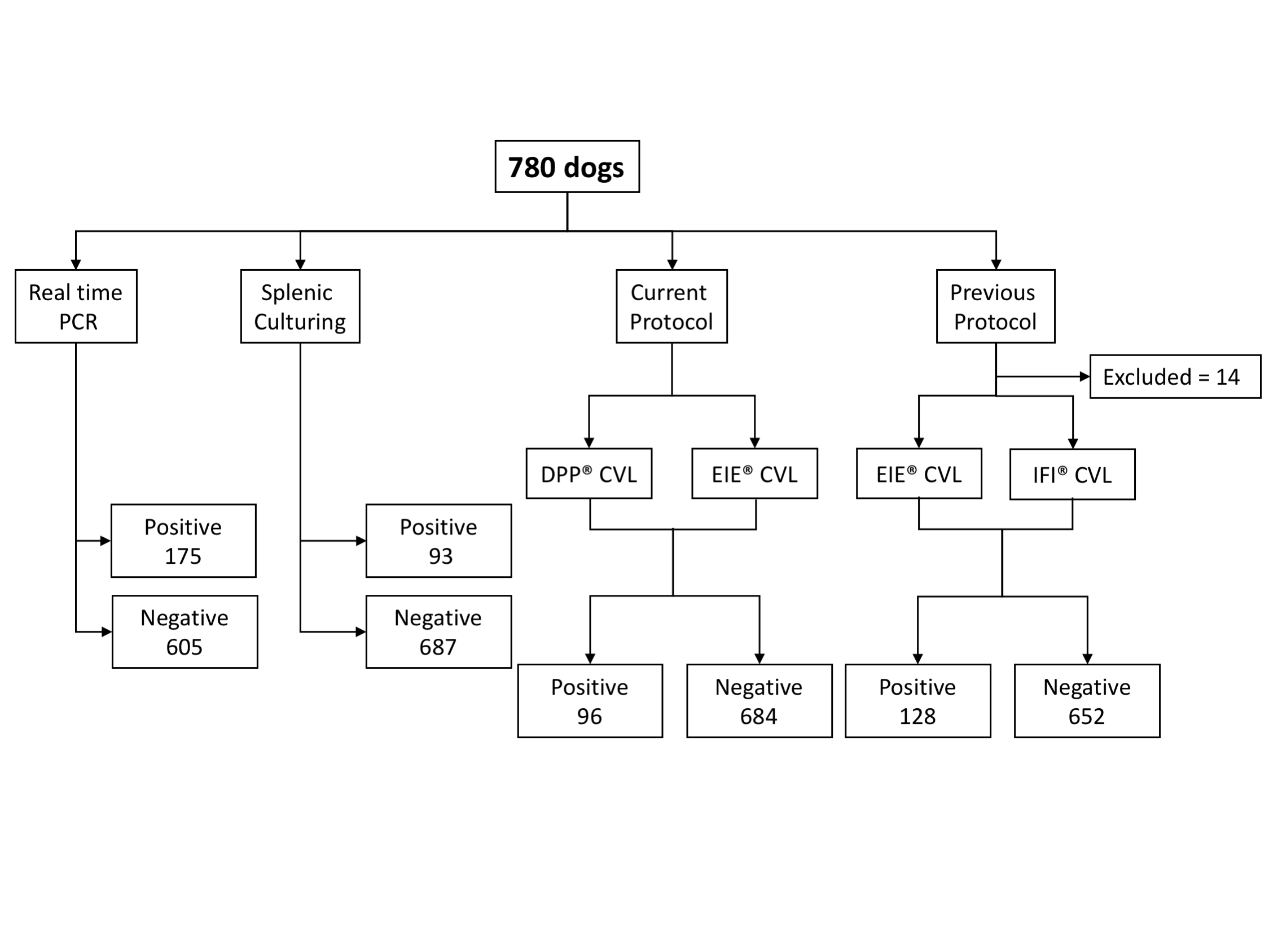

Supplement: S1 Fig — Standards for the Reporting of Diagnostic Accuracy Studies (STARD) description of the experimental design to calculate accuracy of CVL serodiagnostic protocols. (TIFF) [file pntd.0004333.s002.tiff]
